# Association of serum irisin levels with postoperative cognitive dysfunction in older patients following total hip or knee arthroplasty: A prospective observational study

**DOI:** 10.1371/journal.pone.0344177

**Published:** 2026-04-10

**Authors:** Hongxia Zhang, Xue Lin, Yue Zhao, Yuting Zou, Wei Pan, Liangying Yi, Yiping Bai, Daiying Zhang

**Affiliations:** 1 Department of Sterile Processing Nursing, West China Second University Hospital, Sichuan University, Chengdu, Sichuan, China; 2 Key Laboratory of Birth Defects and Related Diseases of Women and Children (Sichuan University), Ministry of Education, Chengdu, Sichuan, China; 3 School of Nursing & Anesthesiology and Critical Care Medicine Key Laboratory of Luzhou, Southwest Medical University, Luzhou, Sichuan, China; 4 Operating room, The Affiliated Hospital, Southwest Medical University, Luzhou, Sichuan, China; 5 Department of Anesthesiology, The Affiliated Hospital, Southwest Medical University, Luzhou, Sichuan, China; Sapienza University of Rome: Universita degli Studi di Roma La Sapienza, ITALY

## Abstract

**Background:**

Postoperative cognitive dysfunction (POCD) is a frequent complication in older patients undergoing hip and knee arthroplasty. While irisin has demonstrated benefits in neurological disorders associated with cognitive impairment, its role in POCD remains unclear. This study investigated the association between serum irisin levels and POCD and assessed the discriminative value of irisin for POCD development.

**Methods:**

This prospective observational study employed Spearman correlation and multifactorial logistic regression analyses to investigate the relationships between these biomarkers and POCD. Receiver operating characteristic analyses were then performed to assess the discriminative value of irisin.

**Result:**

POCD occurred in 37 (31.36%) patients. Serum irisin levels were markedly reduced in patients with POCD relative to those without POCD. T0 irisin (*rho* = 0.675) levels correlated positively with Montreal cognitive assessment-Basic (MoCA-B) scores. Logistic regression analysis identified T0 irisin, T1 irisin, T0 Tumor necrosis factor-α (TNF-α), and hypertension as independent factors associated with the development of POCD (all *P* < 0.05). T0 irisin showed significant discriminative value for POCD, with a cutoff value of 302.840 ng/mL (Area Under the Curve [*AUC*]: 0.817; sensitivity: 0.757; specificity: 0.765).

**Conclusion:**

Irisin may serve as an early warning biomarker for POCD, offering new insights for its prevention and treatment.

## Introduction

Postoperative cognitive dysfunction (POCD) is a neurological complication that extends beyond the acute recovery period following surgery and anesthesia [1]. It frequently involves multiple cognitive domains, with memory and executive function most affected [2]. Clinically, POCD is characterized by impaired memory, learning difficulties, reduced attention span, personality changes, and abnormal social behavior [3]. The postoperative cognitive impairment referred to in this study specifically denotes a cognitive state where the MoCA-B score, when retested one week after hip or knee replacement surgery, shows a decrease of ≥2.88 points (i.e., ≥ 1 standard deviation) compared to the preoperative baseline. This condition is relatively common among older patients undergoing hip and knee arthroplasty, with an incidence rate of 31.34% [4].

The presence of POCD correlates with an increased risk of disease progression and adverse outcome [5]. Specifically, the one-year mortality rate in patients with POCD is approximately double that of patients without the condition within 3 months post-surgery [6]. Furthermore, the occurrence of POCD increases the likelihood of developing Alzheimer’s disease (AD), potentially resulting in permanent brain damage and a deterioration in the quality of life of older patients with a fragile central nervous system [7]. This, in turn, imposes a substantial health and economic burden on patients, healthcare systems, and society [8].

Neuroinflammation is a crucial process in the pathogenesis of POCD, with serum pro-inflammatory cytokines including Interleukin-6 (IL-6), Interleukin-1β (IL-1β), and Tumor Necrosis Factor-α (TNF-α) are directly implicated [9,10]. During the perioperative period, the release of TNF-α and IL-1 activates the Nuclear Factor κB (NF-κB) pathway, upregulating cellular Cyclo-oxygenase 2 (COX-2) in neurovascular endothelial cells. This process promotes prostaglandin synthesis, disrupts the blood-brain barrier, and allows peripheral inflammatory factors to diffuse into the central nervous system, thereby inducing central nervous system inflammatory responses [11]. The hippocampus, due to its extensive expression of IL-6 and IL-1β receptors, is particularly vulnerable to damage from microglia-released pro-inflammatory cytokines under pathological conditions [12]. Over-activation of microglia can trigger a neurotoxic response, resulting in hippocampal neuronal damage, impaired synaptic function, and, subsequently, cognitive decline [13].

Irisin, an actin-cleaved and fibronectin type III structural domain-containing protein 5 released into the peripheral circulation, has been shown to enhance adult hippocampal neurogenesis and synaptic plasticity in the hippocampus’s dentate gyrus [14]. This occurs through the stimulation of the hippocampus’s production of Brain-derived neurotrophic factor (BDNF) and acts as a mediator of multiple inflammatory pathways, mitigating the progression of neuroinflammation [15]. This contributes to neuroprotection and cognitive improvement [16]. Animal studies have highlighted the neuroprotective potentials of irisin in various ischemic and neurodegenerative conditions, including AD, demonstrating its remarkable clinical value. In mouse models of ischemic stroke, irisin reduces postischemic neuronal injury, inflammation, and hippocampal apoptosis, thereby providing neuroprotection [17]. Similarly, in AD mouse models, irisin protects the hippocampus by increasing the secretion of neutral endopeptidase from astrocytes, reducing amyloid-beta deposition [18].

Although irisin has demonstrated multiple neuroprotective effects in animal models, based on current research evidence, its role in perioperative neurocognitive disorders in humans remains unclear. The purpose of this study was to assess the relationship between serum irisin levels and POCD as well as the discriminative value of irisin in determining the risk of developing postoperative cognitive dysfunction.

## Methods

### Participants

This prospective observational clinical study followed the STROBE reporting guidelines for observational studies. Using convenience sampling, older patients undergoing their first elective hip or knee arthroplasty under general anesthesia at the Affiliated Hospital of Southwest Medical University between April 2022 and April 2023 were systematically recruited. The inclusion criteria are age ≥ 60 years, first-time total hip or knee arthroplasty under general anesthesia, normal coagulation profile, and American Society of Anesthesiologists (ASA) classification grades I–III. The exclusion criteria include preoperative MoCA-B score <19 points, mental dysfunction or use of antipsychotic medications, and comorbid conditions affecting normal irisin secretion (e.g., chronic renal failure, hypothyroidism, primary myopathies or neuromuscular diseases such as muscular dystrophy and myasthenia gravis, and neurodegenerative diseases) [16]. Others are cardiac, hepatic, or renal insufficiency; malignant tumors; inability to cooperate with assessments; serious adverse events resulting in physical damage, functional impairment, or life-threatening conditions (e.g., surgical site infection, prosthesis dislocation); and incomplete cognitive function assessments.

The study was authorized by the Southwest Medical University Affiliated Hospital’s Ethics Committee (Ethics Approval No. KY2022103) and complied with the Declaration of Helsinki. The China Clinical Trial Center has registered this study (ChiCTR2300075455). All participants gave their informed consent.

### Observation indicators

The following data were collected:

(1) Demographic characteristics, including gender, age, education level, and body mass index (BMI).
(2) Clinical indicators, such as a history of hypertension, diabetes mellitus, or hyperlipidemia; MoCA-B score; surgery duration; anesthesia duration; blood loss; and infusion volume.
(3) Laboratory indicators, including serum levels of irisin, BDNF, IL-1β, IL-6, and TNF-a levels.

### Laboratory tests

3 milliliters of fasting venous blood were drawn from patients in the early morning 1 day before surgery (T0) and 1 week after surgery (T1). Samples were immediately stored at 2–8℃ temporarily and then centrifuged at 3000 rpm for 15 min. The supernatant (serum) was extracted into a freezing tube, labeled, and stored at −80°C in a medical cryostat until analysis to avoid repeated freeze-thaw cycles.

Serum irisin, BDNF, IL-1β, IL-6, and TNF-a levels were measured using enzyme-linked immunosorbent assay (ELISA) kits (Chengdu YuanNuoTianCheng Science and Technology Co.). Batch numbers were as follows: human serum irisin (YMS1912-A), BDNF (YMS0027-A), IL-1β (YMS0179-A), IL-6 (YMS0049-A), and TNF-a (YMS0121-A). All kits have received approval from the State Food and Drug Administration.

### Anesthesiology

All patients received general anesthesia. Before induction, vital signs were monitored, and oxygen was administered at 6 L/min. Anesthesia was induced with sufentanil (0.3–0.4 ug/kg), isoproterenol (1.5–2.0 mg/kg), and cis-atracurium (0.2–0.3 mg/kg). Intraoperatively, the infusion rate of anesthetic agents was adjusted to maintain a bispectral index between 40 and 60. Blood pressure and heart rate were maintained within ±20% of baseline values, and vasoactive drugs were administered as needed. Thirty minutes before the end of the procedure, intravenous diazoxide (5 mg) and granisetron (3 mg) were administered. Postoperatively, patients received patient-controlled intravenous analgesia (PCIA), consisting of 210 mL saline, 150 ug sufentanil, and 4 mg butofenol, for postoperative paroxysms, with an initial bolus dose of 10 mL and a self-controlled dose of 0.5 mL.

### Cognitive function assessment

Patients’ overall cognitive function was assessed using the MoCA-B at 8:00 a.m., 1 day preoperatively and 1 week postoperatively. The MoCA-B includes 11 items from eight cognitive domains: executive function, memory, language, orientation, computation, abstraction, visual perception, and attention.

Assessors were certified through the official MoCA website (www.mocatest.org) and were trained to administer, score, and interpret the tests. A decrease of ≥2.88 points in the MoCA-B score from the preoperative baseline (i.e., ≥ 1 standard deviation [*SD*]) was referred to as POCD [1]. Participants were categorized into POCD and non-POCD groups based on MoCA-B scores.

### Sample size calculation

Receiver operating characteristic (*ROC*) curve was used to determine the sample size. Preliminary data from 20 patients suggested an expected area under the curve (*AUC*) for irisin of 0.88 and an expected POCD incidence of 30% at 1 week postoperatively. With a two-tailed alpha of 0.05, power (*1-β*) of 0.90, and a 20% anticipated loss to follow-up, this study required at least 28 patients with POCD and 65 patients without POCD.

### Statistical analysis

Statistical analyses were conducted utilizing Statistical Package for the Social Sciences 26.0 (SPSS Inc., Armonk, NY, USA), R software (v4.2.3, R Core Team 2023), and GraphPad Prism 8.0 (GraphPad Inc., San Diego, CA, USA) for visualizations.

The Kolmogorov-Smirnov test was used to evaluate the normality of the data. The independent samples t-test was utilized to compare normally distributed data, which were represented as mean ± standard deviation ($\overset{―}{x}\pm s$). The Mann-Whitney U rank-sum test was used to compare non-normally distributed data, which were represented as a median and interquartile range [*M* (*P25*, *P75*)]. The *χ*^2^ test was used to examine categorical data, which were displayed as frequencies or percentages.

Spearman’s rank correlation was used to conduct correlation analysis. Univariate logistic regression was used to identify risk factors for POCD, and variables with *P* < 0.05 were added to a multivariate logistic regression model. *ROC* curves were plotted to assess the discriminative value of irisin for POCD risk. *P* value <0.05 was regarded as statistically significant. Missing values (≤5%) were imputed using the median for continuous variables and the mode for categorical variables [19].

## Results

### Patient characteristics

This study initially included 180 older patients undergoing hip and knee arthroplasty. Based on the exclusion criteria, 7 individuals with preoperative MoCA-B scores <19 points, 13 patients with mental dysfunction or on antipsychotic medications, 4 patients with concomitant diseases affecting normal irisin secretion, 5 patients with cardiac, hepatic, or renal insufficiency, 8 patients with tumors, and 6 patients unable to cooperate with the assessment were excluded. The study cohort was initially comprised of 137 patients. During follow-up, 3 patients experienced serious postoperative complications, 12 patients were lost to follow-up due to early discharge and other reasons, and 4 blood specimens were deemed inadequate. The statistical analysis ultimately comprised 118 patients.

Among these 118 patients, 37 (31.36%) developed POCD 1 week after surgery. Comparison of general characteristics between patients in the POCD and non-POCD groups revealed no statistically significant differences in gender, age, BMI, diabetes history, hyperlipidemia history, duration of surgery, duration of anesthesia, blood loss, and fluid transfusion (*P* > 0.05). However, patients with POCD had significantly lower education levels (*P* = 0.038) and a higher prevalence of hypertension (*P* = 0.018) compared to the non-POCD group. (Table 1)

**Table 1:** Patient demographics and clinical characteristics.

Characteristics	Total(n = 118)	POCD(n = 37)	Non-POCD (n = 81)	*P-value*
Gender(n, %)				
Male	36 (30.50)	11 (29.73)	25 (30.86)	0.901
Female	82 (69.50)	26 (70.27)	56 (69.14)	
Age(years)	69.00 (65.00–74.00)	69.00 (64.00–75.00)	70.00 (65.00–73.50)	0.855
Education (years)	6.00 (0.00–6.00)	6.00 (0.00–6.00)	6.00 (6.00–9.00)	0.038
BMI(Kg/m^2)^	24.20 (21.76–26.21)	24.00 (20.85–25.95)	24.40 (22.37–26.34)	0.313
Hypertension (n, %)	64 (54.20)	26 (70.30)	38 (46.90)	0.018
Diabetes(n, %)	26 (22.00)	7 (18.90)	19 (23.50)	0.581
Hyperlipidemia (n, %)	56 (47.50)	16 (43.20)	40 (49.40)	0.384
Duration of surgery (min)	115.00 (90.00–140.00)	115.00 (92.50–155.00)	115.00 (90.00–135.00)	0.388
Duration of anesthesia(min)	170.00 (150.00–210.00)	170.00 (150.00–210.00)	170.00 (142.50–215.00)	0.657
Bleeding loss(ml)	200.00 (100.00–300.00)	200.00 (100.00–300.00)	200.00 (100.00–300.00)	1.000
Total infusion(ml)	1500.00 (1100.00–1700.00)	1400.00 (1100.00–1750.00)	1500.00 (1100.00–1700.00)	0.746
T0 Irisin(ng/ml)	322.15 ± 51.56	282.96 ± 33.28	340.06 ± 48.53	<0.001
T0 BDNF(ng/ml)	10.09 ± 1.78	9.99 ± 1.78	10.14 ± 1.78	0.662
T0 IL-6(pg/ml)	23.69 ± 4.40	24.37 ± 5.08	23.38 ± 4.04	0.258
T0 IL-1β(pg/ml)	49.70 ± 8.59	49.16 ± 9.67	49.95 ± 8.10	0.648
T0 TNF-α(pg/ml)	39.96 ± 10.60	42.99 ± 12.00	38.57 ± 9.66	0.035
T1 Irisin(ng/ml)	285.71 ± 51.66	261.43 ± 47.99	296.80 ± 49.69	<0.001
T1 BDNF(ng/ml)	8.66 ± 2.21	8.15 ± 2.33	8.90 ± 2.12	0.087
T1 IL-6(pg/ml)	27.91 ± 5.99	30.31 ± 6.76	26.82 ± 5.29	0.003
T1 IL-1β(pg/ml)	52.83 ± 10.31	53.08 ± 10.03	52.72 ± 10.52	0.861
T1 TNF-α(pg/ml)	43.80 ± 11.98	47.37 ± 12.91	42.17 ± 11.24	0.028

**Fig 1:**
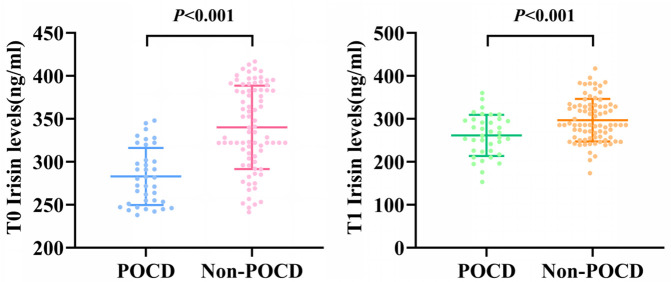
Comparison of T0 irisin and T1 irisin levels between POCD and non-POCD groups(a for T0, b for T1).

Serum biomarker analysis revealed substantial differences between the two groups in T0 irisin, T0 TNF-α, T1 irisin, T1 IL-6 and T1 TNF-α levels (*P* < 0.05). T0 irisin and T1 irisin levels were significantly lower in the POCD group than in the non-POCD group (*P* < 0.001, Fig 1).

### Correlation analysis

**Fig 2:**
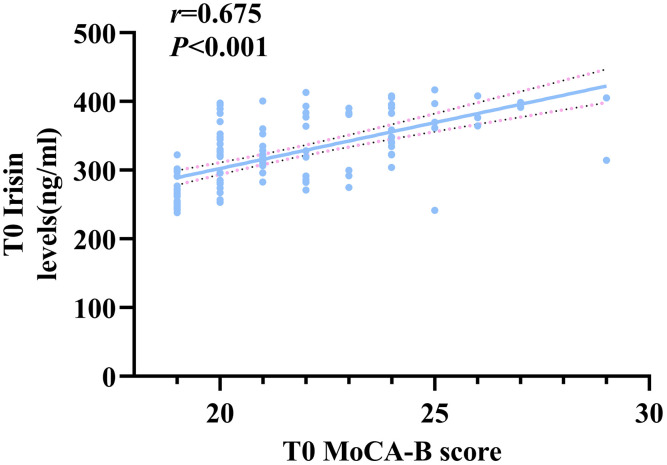
The Correlation of T0 Irisin levels and T0 MoCA-B scores.

The associations between MoCA-B scores and preoperative and postoperative serum irisin, BDNF, IL-6, IL-1β, and TNF-α levels were assessed using Spearman correlation analysis. T0 irisin levels were significantly positively correlated with MoCA-B scores (*rho* = 0.675, *P* < 0.001), while T1 irisin and T1 IL-6 levels (*rho* = −0.185, *P* = 0.045) were weakly correlated with MoCA-B scores (*rho* = 0.261, *P* = 0.004, Fig 2). However, T0 IL-6, IL-1β, and TNF-α levels, as well as T1 BDNF and IL-1β levels had no discernible relationship to MoCA-B scores (*P* > 0.05).

### Logistic regression

Before constructing a multivariate logistic regression model, we assessed multicollinearity among continuous and categorical variables using the variance inflation factor (*VIF*). Using a threshold of *VIF* < 5, we observed no significant multicollinearity among all variables included in the final model (S1 Table in S1 File). To determine whether serum irisin levels were independently associated with the POCD development, variables with *P* < 0.05 in the univariate logistic regression analysis were included in a multivariate logistic regression model (S2 Table in S1 File). The results showed that T0 irisin (*OR*: 0.971, 95% *CI*: 0.958–0.984, *P* < 0.001), T1 irisin (*OR*: 0.983, 95% *CI*: 0.970–0.997, *P* = 0.014), T0 TNF-α (*OR*: 1.074, 95% *CI*: 1.012–1.139, *P* = 0.019) and hypertension (*OR*: 3.573, 95% *CI*: 1.132–11.049, *P* = 0.030) were independently associated with POCD development (Table 2).

**Table 2:** Multivariate logistic regression analysis.

Parameters	*OR*	95%*CI*	*P-value*
Education (years)	0.885	0.742–1.055	0.173
Hypertension	3.573	1.132–11.049	0.030
T0 irisin (ng/ml)	0.971	0.958–0.984	<0.001
T0 TNF-α (pg/ml)	1.074	1.012–1.139	0.019
T1 irisin (ng/ml)	0.983	0.970–0.997	0.014
T1 IL-6 (pg/ml)	1.093	0.984–1.215	0.096
T1 TNF-α (pg/ml)	1.021	0.975–1.068	0.376

### ROC curve analysis

**Fig 3:**
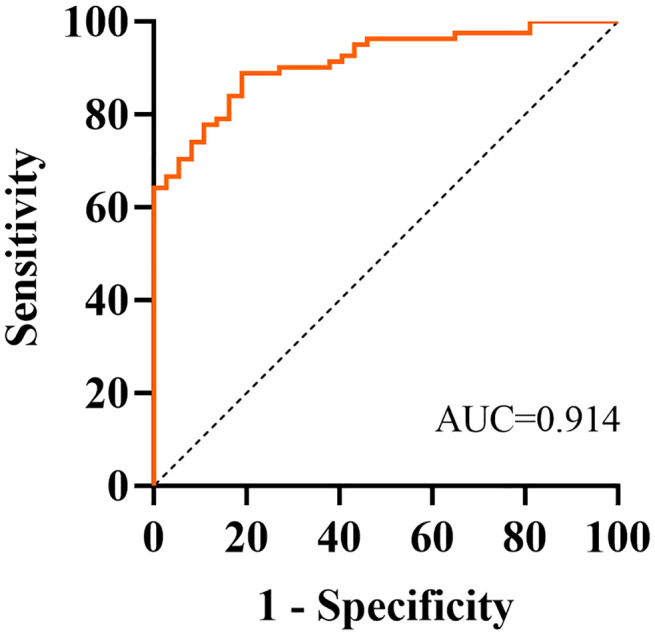
ROC curve for joint prediction.

Based on the results of the multivariate regression analysis, Receiver Operating Characteristic (ROC) curve analysis was conducted to evaluate the discriminative value of education levels, hypertension, T0 irisin, T0 TNF-α, T1 irisin, T1 IL-6, and T1 TNF-α levels for POCD. Education levels, hypertension, T0 irisin, T0 TNF-α, T1 irisin, T1 IL-6, and T1 TNF-α were all significant predictors of POCD (*P* < 0.05). T0 irisin had a significant discriminative value for POCD with a cutoff value of 302.840 ng/ml(AUC: 0.817, sensitivity: 0.757, specificity: 0.765). Combining the seven biomarkers further enhanced the discriminative accuracy, yielding an AUC of 0.914 (95%CI:0.865 ~ 0.963, *P* < 0.001), sensitivity of 0.811, and specificity of 0.889 (Table 3, Fig 3).

**Table 3:** ROC curve analysis results.

Parameters	AUC	95%*CI*	*P-value*	cut-off value	sensitivity	specificity
Education (years)	0.636	0.531–0.741	0.018	–	0.405	0.790
Hypertension	0.617	0.509–0.725	0.042	–	0.703	0.531
T0 Irisin	0.817	0.741–0.893	<0.001	302.840	0.757	0.765
T0 TNF-α	0.613	0.494–0.732	0.050	48.050	0.432	0.864
T1 Irisin	0.681	0.579–0.783	0.002	316.675	0.919	0.383
T1 IL-6	0.643	0.529–0.756	0.013	29.725	0.541	0.765
T1 TNF-α	0.629	0.514–0.744	0.025	44.510	0.649	0.659
**combination**	**0.914**	**0.865–0.963**	**<0.001**	**–**	**0.811**	**0889**

## Discussion

In this study, 37 patients developed POCD 1 week after hip and knee arthroplasty, yielding an incidence rate of 31.36%, which aligns with findings from previous research [1]. Serum irisin is independently associated with POCD occurrence, exhibiting strong discriminative value. In addition, patients with hypertension and high preoperative TNF-α levels were more susceptible to POCD.

The diagnosis of POCD currently depends predominantly on neuropsychological testing [20]. MoCA-B is specifically designed to screen for cognitive dysfunction in illiterate and poorly educated older adults, with its validity established in the Chinese older population [21]. Considering that the majority of participants in this study were elderly patients and had less than 6 years of formal education, the Chinese version of MoCA-B was chosen to evaluate the overall cognitive function [22]. In this study, we defined POCD as a MoCA-B score that was one standard deviation (SD) lower than the preoperative score. While this method is widely accepted for diagnosing POCD, it has some limitations [23]. First, it is primarily applicable to cognitive function assessment conducted within 1–7 days postoperatively, limiting its utility for longer-term follow-up [24]. Second, this approach relies on the SD calculated from preoperative scores, which can vary across studies, making it difficult to directly compare POCD incidence rates between different cohorts [25]. Additionally, factors such as patient education level, examiner skill, and environmental changes can affect the stability of cognitive assessment results, thereby increasing test-retest variability [26]. Irisin is an objective, quantifiable blood marker closely associated with the occurrence of POCD, offering promising potential as a crucial biological basis for its diagnosis. When combined with neuropsychological scales that are more subjective in nature, it can comprehensively enhance the accuracy and reliability of diagnosis.

Our findings additionally demonstrated that serum irisin levels were independently correlated with the onset of POCD. Irisin can cross the blood-brain barrier and induce the expression of BDNF and other neuroprotective genes in the hippocampus, contributing to the cognitive benefits of exercise [27]. Previous studies have documented serum irisin levels in individuals with neurological disorders such as AD [28], Parkinson’s disease [29] and stroke [30] — conditions commonly associated with cognitive impairment. Additionally, irisin levels appear to decline progressively with the severity of these diseases. Serum irisin levels have also been proposed as an early predictor of cognitive decline in patients with AD [28] and vascular dementia [31]. The primary clinical significance of irisin as a potential biomarker lies in its value for preoperative screening. By measuring baseline irisin levels, clinicians may identify patients at high risk for POCD [32]. This enables vulnerable individuals to promptly benefit from enhanced intraoperative monitoring, optimized anesthesia protocols, and early neuroprotective interventions—shifting clinical practice from reactive postoperative management to proactive preoperative prevention [33]. Additionally, dynamic monitoring of irisin levels in the early postoperative period provides objective insight into the progression of neurological injury and repair [34]. Trends in irisin levels offer greater prognostic value than single-time-point measurements, supporting risk re-stratification and helping identify patients who may require intensified postoperative interventions [35].

In this study, patients with hypertension were found to be more susceptible to POCD. Furthermore, the correlation between hypertension and cognitive function was more pronounced in people aged 60 years and older [22]. When hypertension coexists with cognitive impairment, it exacerbates the risk of all-cause mortality and cardiovascular disease-specific mortality in older patients [36]. Among older patients with cognitive impairment, comorbid hypertension elevated the probability of death from all causes 2.73-fold (95% CI: 1.78–4.17) and cardiovascular disease-specific mortality by 5.3-fold (95% CI: 2.54–11.04) [37]. Thus, hypertension significantly increases cognitive risk and disease burden in the perioperative older population. In various neurodegenerative diseases, TNF-α not only accelerates disease progression by promoting inflammatory responses, but may also cause hippocampal neuronal damage and synaptic dysfunction, resulting in cognitive impairment [38]. Irisin improves cognition primarily by suppressing neuroinflammation [11]. Irisin binds with integrin αVβ5 receptors on microglia, decreasing the production and release of pro-inflammatory cytokines like TNF-α, IL-1β, and IL-6, thereby markedly inhibiting neuroinflammation [39]. Additionally, irisin modulates pro-inflammatory microglia into non-inflammatory or anti-inflammatory phenotypes through activation of the ERK-CREB pathway by BDNF and its receptor, tropomyosin receptor kinase B (TrkB). This mechanism suppresses nuclear factor-κB (NF-κB) activation, further reducing inflammation [40]. Irisin’s ability to suppress pro-inflammatory factors, such as IL-6 and TNF-α, highlight its potential in mitigating postoperative cognitive dysfunction.

Exercise has been recognized as an effective strategy to promote irisin secretion. For instance, serum levels triple following 10 weeks of regular endurance exercise in humans [41], while plasma irisin concentrations markedly increase by 65% in mice after 3 weeks of free-round exercise [42]. Irisin crosses the blood-brain barrier and activates neuroprotective genetic pathways in the hippocampus, stimulating BDNF secretion from microglia and astrocytes, thereby improving hippocampal-dependent spatial learning and memory [43]. BDNF is both neuroprotective and anti-inflammatory, serving as a key regulator of hippocampal neurogenesis [27]. Research indicates that engaging in regular exercise lowers the risk of dementia by 28% and AD by 45%, respectively, and that being physically active is closely linked to a lower risk of AD [44]. The cognitive benefits of exercise are particularly pronounced in older adults and patients with neurodegenerative diseases. For individuals aged 70–80 years, consistent exercise reduces the risk of AD by approximately 40% compared to sedentary peers [45]. Emerging evidence indicates that exercise-induced cognitive improvement may be driven by mechanisms beyond physical health improvements, although these mechanisms have not yet been fully elucidated [46]. Irisin, an actin-induced protein secreted in response to physical activity, has been preliminarily associated with POCD in this study. Therefore, we hypothesize that irisin may play a key regulatory role in exercise-mediated cognitive improvement, providing a theoretical basis and directional guidance for future research.

## Conclusion

Serum irisin levels are closely associated with POCD and hold significant discriminative value. Irisin may serve as an early warning biomarker for POCD, offering new insights for its prevention and treatment. However, this study has some limitations. First, it is a single-center observational study involving older patients undergoing total hip or knee arthroplasty under general anesthesia. Therefore, the generalizability and applicability of the findings are limited, and caution is warranted when extrapolating results to other populations or anesthesia modalities. Second, the current research remains in an exploratory and observational phase. Intraoperative and postoperative parameter recording was not sufficiently detailed or comprehensive. Although multivariate logistic regression was employed to control for confounding factors, unmeasured confounders (e.g., perioperative medication, intraoperative hypotension, oxygenation, pain) may have inevitably influenced the observed outcomes. This study retains the term “POCD” to highlight the research value of the first week post-surgery as an independent cognitive assessment window in early rehabilitation, while maintaining data comparability with previous classic studies. This decision is also based on the distinct clinical characteristics of cognitive deficits during this phase compared to postoperative delirium (POD). It should be noted that this nomenclature differs from the “delayed neurocognitive recovery (DNCR)” recommended in current guidelines. The diagnostic criteria for POCD also remain controversial. Our approach—diagnosing based on a MoCA-B score reduced by one SD from preoperative levels—has limitations, including short follow-up duration and high test-retest variability. Furthermore, this study focused exclusively on acute postoperative POCD events, lacking long-term neurocognitive follow-up data. Additionally, the use of multiple imputation to handle missing data led to overlapping data points in the relevant graphs, potentially affecting the intuitive interpretation of data distribution. Future large-scale, multicenter clinical and basic research building upon this study could further elucidate the beneficial effects of irisin in the development of POCD.

## Supporting information

S1 File **S1 Table**. Collinearity Statistics. **S2 Table**. Univariate regression analysis.
(ZIP)
