# Supplementary material for: Association of serum irisin levels with postoperative cognitive dysfunction in older patients following total hip or knee arthroplasty: A prospective observational study
Source: PLoS One. 2026 Apr 10;21(4):e0344177. doi: 10.1371/journal.pone.0344177 (PMC13068216; doi:10.1371/journal.pone.0344177)
Supplement: S1 File — S1 Table. Collinearity Statistics. S2 Table. Univariate regression analysis. (ZIP) [file pone.0344177.s001.zip › Supporting information/S1_Table.pdf]

S1 Table Collinearity Statistics

Parameters	Standardized Coeficients Beta	Collinearity Statistics	
		Tolerance	VIF
Education	-0.127	0.933	1.071
Hypertension	0.189	0.943	1.060
T0 Irisin	-0.413	0.855	1.169
T0 TNF-α	0.157	0.915	1.093
T1 Irisin	-0.170	0.891	1.122
T1 IL-6	0.109	0.818	1.223
T1 TNF-α	-0.059	0.863	1.159
