# Supplementary material for: Association of serum irisin levels with postoperative cognitive dysfunction in older patients following total hip or knee arthroplasty: A prospective observational study
Source: PLoS One. 2026 Apr 10;21(4):e0344177. doi: 10.1371/journal.pone.0344177 (PMC13068216; doi:10.1371/journal.pone.0344177)
Supplement: S1 File — S1 Table. Collinearity Statistics. S2 Table. Univariate regression analysis. (ZIP) [file pone.0344177.s001.zip › Supporting information/S2_Table.pdf]

S2 Table Univariate regression analysis

**Parameters**	***OR***	**95%*CI***	***P-value***
Gender	1.055	0.452~2.464	0.901
Age	1.009	0.956~1.066	0.732
Education	0.873	0.776~0.981	0.023
BMI	0.942	0.842~1.054	0.299
Hypertension	2.675	1.167~6.128	0.020
Diabetes	0.761	0.289~2.009	0.582
Hyperlipidemia	0.781	0.357~1.708	0.536
Duration of surgery	1.005	0.997~1.013	0.231
Duration of anesthesia	1.003	0.994~1.011	0.502
Bleeding loss	1.000	0.998~1.003	0.759
Total infusion	1.000	0.999~1.001	0.725
T0 Irisin	0.971	0.960~0.983	<0.001
T0 BDNF	0.952	0.764~1.186	0.659
T0 IL-6	1.054	0.963~1.153	0.256
T0 IL-1β	0.989	0.945~1.035	0,645
T0 TNF-α	1.042	1.002~1.084	0.038
T1 Irisin	0.985	0.976~0.994	0.001
T1 BDNF	0.855	0.713~1.024	0.089
T1 IL-6	1.105	1.032~1.184	0.004
T1 IL-1β	1.003	0.966~!.042	0.860
T1 TNF-α	1.038	1.003~1.074	0.031
